# Multimodality Imaging of the Peripheral Venous System

**DOI:** 10.1155/2007/54616

**Published:** 2006-12-24

**Authors:** Diana Gaitini

**Affiliations:** Unit of Ultrasound, Department of Medical Imaging, Rambam Medical Center and Faculty of Medicine, Technion–Israel Institute of Technology, P.O. Box 9602, Haifa 31096, Israel

## Abstract

The purpose of this article is to review the spectrum of image-based diagnostic tools used in the investigation of suspected deep vein thrombosis (DVT). Summary of the experience gained by the author as well as relevant publications, regarding vein imaging modalities taken from a computerized database, was reviewed. The imaging modalities reviewed include phlebography, color Doppler duplex ultrasonography (CDDUS), computerized tomography angiography (CTA) and venography (CTV), magnetic resonance venography (MRV), and radionuclide venography (RNV). CDDUS is recommended as the modality of choice for the diagnosis of DVT. A strategy combining clinical score and D-dimer test refines the selection of patients. Phlebography is reserved for discrepant noninvasive studies.

## 1. INTRODUCTION

Deep venous thrombosis (DVT) of the lower limb is a common and life-threatening condition. The incidence in the United States is estimated at 70–100 000 new cases/year with as many as 200 000 hospitalizations/year. It carries a risk of pulmonary embolism (PE) and the development of post-thrombotic syndrome. The incidence of PE is calculated at 600 000 cases/year, 100 000 of them are fatal [1–3]. Risk factors for lower extremity acute venous occlusion range from, prolonged immobilization to hypercoagulability syndromes, trauma, and malignancy. Venous thromboembolism (VTE) is a leading cause of morbidity and mortality during pregnancy and puerperium, and is second only to hemorrhage as the commonest cause of maternal death during pregnancy [4]. There is a fivefold increased risk of VTE in pregnant women compared with nonpregnant women of a similar age [5].

Malignancy and central venous lines are major risk factors of upper extremity thrombosis (UEDVT) with predicted poor survival. The increasing use of indwelling central venous catheters (CVC) for transparietal feeding, fluid administration, and chemotherapy has resulted in an increased prevalence of upper extremity venous thrombosis, although, the rate of catheter-associated thrombosis decreased in recent years thanks to improvement in biocompatibility and better insertion and maintenance techniques [6]. UEDVT may be asymptomatic or the clinical manifestations are not specific, presenting with arm or neck swelling or pain. In more than half of the cases objective methods of examination are negative for thrombosis. Pulmonary embolism secondary to UEDVT, sometimes a lethal complication, is not unusual and has been reported in a comparable prevalence to lower extremity thrombosis. Other significant complications of UEDVT are loss of vascular access, superior vena cava syndrome, and postthrombotic venous insufficiency [7–9].

The clinical complications (from postthrombotic syndrome to fatal pulmonary embolism) as well the risk of anticoagulant treatment require a precise diagnosis of DVT. The clinical diagnosis is unreliable: only 20–30% of symptomatic patients have proven DVT and 90% of fatal PE are asymptomatic for DVT [10]. Objective methods of examination are demanded to reach an accurate diagnosis. Phlebography, computerized tomography angiography followed by venography (CTA-CTV) and radionuclide venography (RNV) are invasive or semi invasive tests. Color Doppler duplex ultrasonography (CDDUS), and magnetic resonance venography (MRV) are noninvasive methods. This paper highlights the potential risks and benefits of each of these techniques and presents the advantages, disadvantages, and accuracy of the different imaging modalities. An appropriate imaging algorithm for the diagnosis of DVT is presented. The use of clinical pretest probability scoring and diagnostic algorithms can help identify patients requiring further investigation for suspected venous thromboembolism (VTE).

## 2. PHLEBOGRAPHY

**Figure 1:**
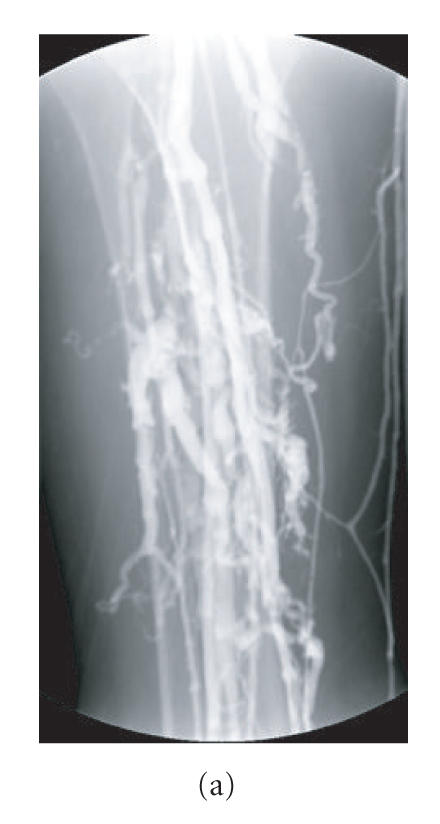

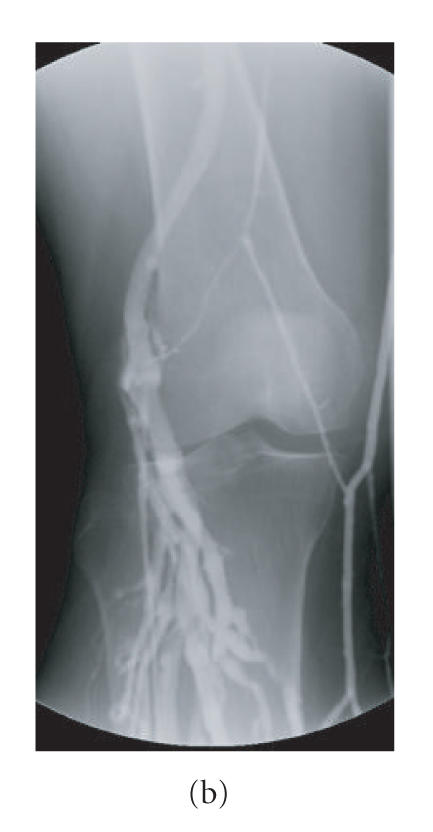

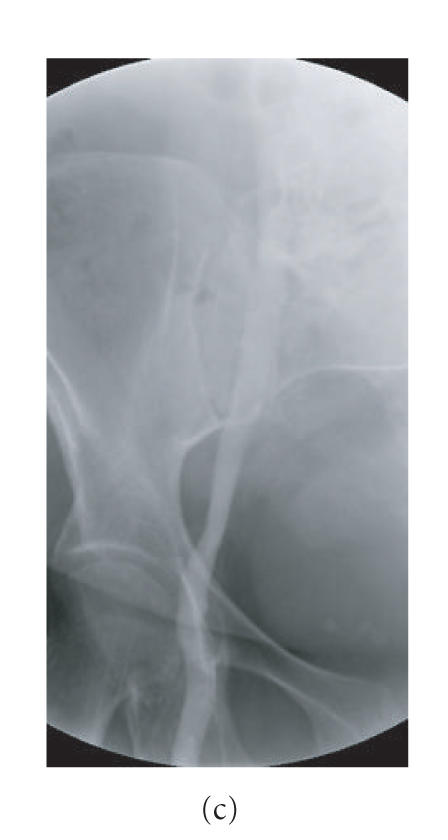
Normal phlebography. (a) Calf veins. (b) Popliteal vein. (c) Femoral vein at the groin and iliac vein at the pelvis.

Phlebography (also called venography, ascending contrast phlebography, or contrast venography) is still considered the gold standard in the diagnosis of peripheral DVT; it is the most accurate test with a nearly 100% sensitivity and specificity [11]. This X-ray examination provides an image of the limb veins after contrast material is injected into a distal vein (Figures 1(a)–1(c)) [12]. Main phlebographic findings are persistent filling defect, abrupt interruption of contrast in a vein, lack of opacification in all or some deep veins, and flow diversion with opacification of collateral branches [13]. Venography relies on the anatomy of the venous system, lacking physiological information. It is painful; expensive, exposes the patient to a fairly high dose of radiation; and can cause complications related to nephrotoxicity and allergic reactions to iodinated contrast agents. It also carries a risk for post venographic phlebitis [14, 15]. In about 5% of cases, there are technical problems in conducting the test. Due to its invasive nature and the risk of complications, it cannot be used neither as a routine test for the diagnosis of symptomatic DVT nor as a screening tool in asymptomatic patients at high risk for DVT. Peripheral phlebography is performed when the noninvasive examination color Doppler US and duplex Doppler is doubtful or technically limited, such in suspected thrombosis of iliac vein, innominate vein, or superior vena cava [13].

## 3. COLOR DOPPLER DUPLEX ULTRASONOGRAPHY

**Figure 2:**
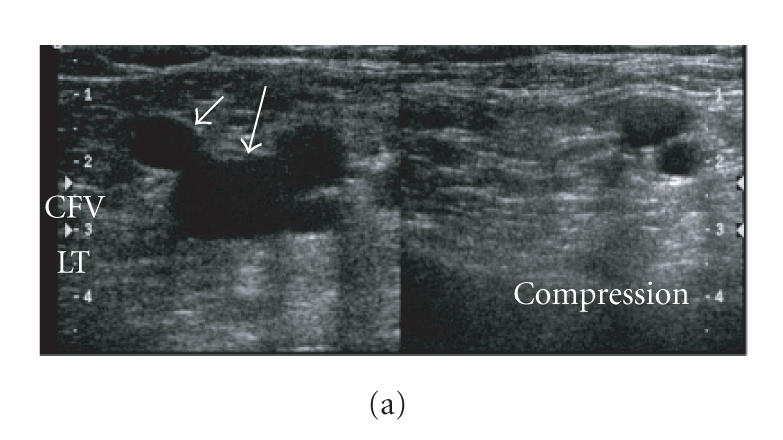

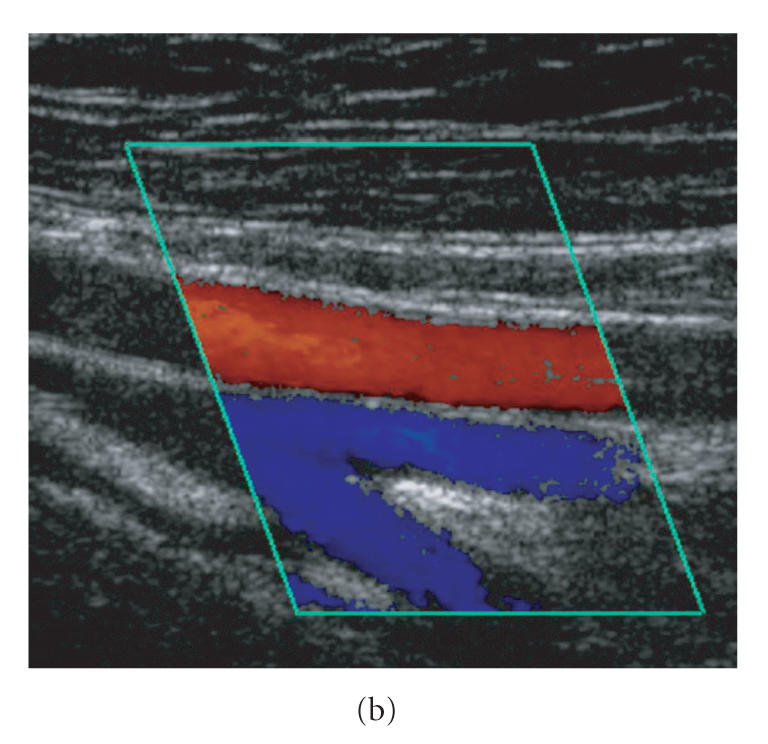

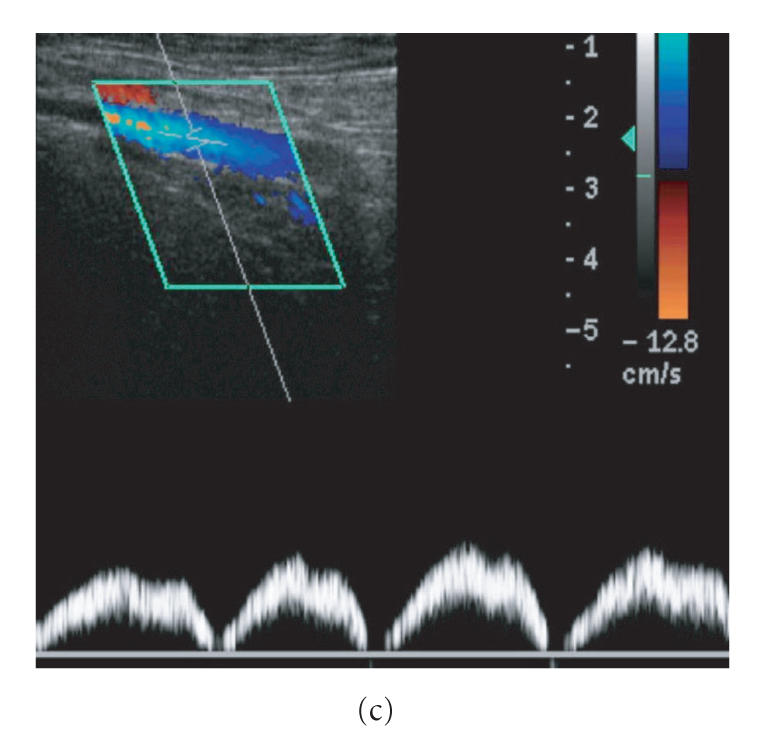
Normal vein. (a) Sonolucent lumen, easily compressible with a slight pressure exerted by the probe. Left side: before compression; right side: during compression; only the arteries remain visible. Large arrow: common femoral vein (CFV); short arrow: great saphenous vein. (b) Flow in femoral artery and veins at the level of the bifurcation. (c) Centripetal nonpulsatile flow in femoral vein, with respiratory phasicity.

**Figure 3:**
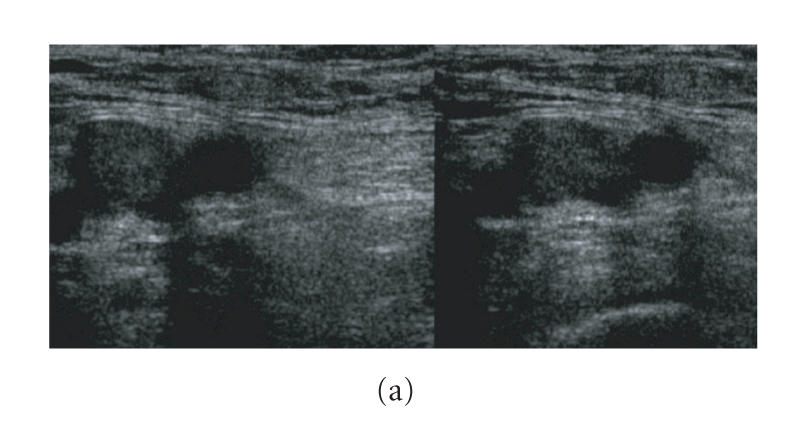

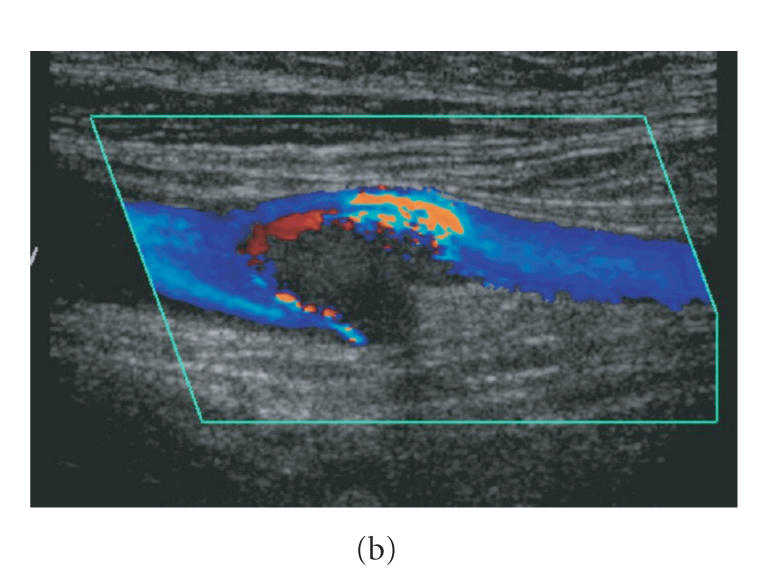

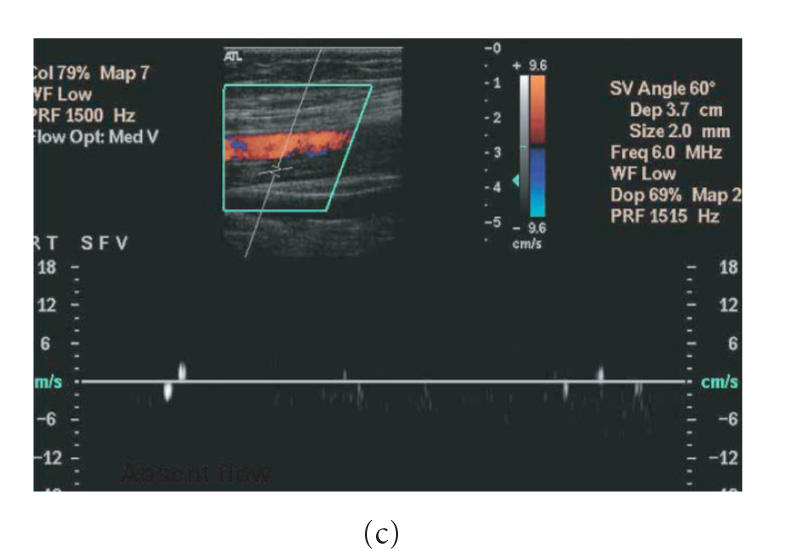
Thrombotic vein. (a) Echogenic lumen, enlarged, noncompressible vein. Left side: before compression; right side: during pressure exerted by the probe, the vein does not collapse. (b) Thrombus at the bifurcation of the femoral vein, seen as color void and turbulent surrounding flow. (c) No flow demonstrated on duplex in a thrombotic femoral vein.

CDDUS is the initial test of choice for diagnosis of acute DVT due to its high accuracy, relatively low cost, portability, widespread, and lack of ionizing radiation [16]. B-mode ultrasound with Doppler color and duplex is the only noninvasive imaging test that combines anatomy and physiology of the veins by visualization of vein morphology and the map of flow velocity and direction. It is required as the primary instrumentation for peripheral venous testing according to the standards of the Intersocietal Commission for the Accreditation of Vascular Laboratories (ICAVL) [17]. CDDUS for the diagnosis of limb vein thrombosis uses a combination of gray-scale, compression, color, and spectral Doppler sonography. Color and spectral Doppler analysis are useful in the diagnostic evaluation of DVT but are best considered as adjuncts to the conventional compression ultrasound examination. The examination is performed by a high-resolution transducer of 7–10 MHz; a lower frequency-4–8 MHz is required for the obese patient, the edematous limb, and the pelvic veins. The veins scanned comprise the deep venous system—femoral vein at the groin and along the thigh, popliteal vein, and tibioperoneal trunk at the upper calf—and the confluence of the superficial great saphenous vein with the femoral vein. The deep calf veins are usually examined when localized pain or swelling is present. CDDUS findings of the normal vein are sonolucent lumen, easily compressible with a slight pressure exerted by the probe and centripetal nonpulsatile flow, with respiratory phasicity and augmentation after Valsalva performance (Figures 2(a)–2(c)). An echogenic lumen, depending on thrombus age, uncompressible and flow devoid is diagnostic of a thrombotic vein (Figures 3(a)–3(c)) [18–24]. The main aim of CDDUS is to confirm or exclude vein thrombosis. Further information includes thrombus extent and characterization—fresh or organized, free floating or attached, and partial or totally occlusive—that have prognostic value for the development of pulmonary embolism and post-thrombotic syndrome. Patients with proximal DVT tend to present a slower and incomplete resolution of thrombus and to develop a more severe post-thrombotic syndrome due to deep venous reflux [25]. Free floating thrombus carries an increased risk of pulmonary embolism, although floating thrombus tends to attach to the vein wall or resolve, not warranting any specific therapeutic procedure [26]. Further diagnostic aims are to detect alternative disorders such as popliteal Baker's cyst, hematoma, aneurysm, pseudoaneurysm, lymphadenopathy, or other tumors, known as “pseudothrombophlebitis,” mimicking DVT. The incidence of these alternative diagnoses is 11–18% [27]. A bilateral examination is indicated when high-risk patients are screened and in the workflow of suspected PE in patients with risk factors for DVT. Due to its high specificity, complete ultrasound examination of the proximal and distal veins at least down to the level of the popliteal trifurcation allows withholding anticoagulant therapy without the risk of major complications. Isolated calf vein thrombosis does not carry a significant adverse outcome; scanning the calf with localized symptoms or physical findings is cost-effective. A repeat examination is warranted if the clinical findings worsen; otherwise, a single examination is enough [28]. The sensitivity and specificity of USD for the diagnosis of DVT in symptomatic patients is very high. Compressibility under probe pressure (CUS) is the most accurate test; for proximal DVT, femoral, and popliteal veins, compression US reached a sensitivity of 97 to 100% and a specificity of 98 to 99%. For isolated calf DVT, the sensitivity dropped to 50–70% and the specificity to 60%. An echogenic lumen has a low sensitivity of about 50% for both proximal and calf DVT, due to the low echogenicity of the fresh thrombus [29–32]. In a meta-analysis of 100 cohort studies that compared Duplex US to contrast venography in patients with suspected DVT; the sensitivity for proximal DVT was 96.5%, for distal calf DVT, 71.2% and specificity of 94.3%; the sensitivity improved in the recent years probably due to equipment development, US technique used, and operator expertise [33].

Ultrasonography is the primary imaging modality also for the diagnosis of upper-extremity thrombosis (UEDVT). The veins examined include the deep system—internal jugular, subclavian, axillary, and brachial veins. The superficial veins—cephalic and basilica—are scanned in case of peripherally inserted catheter-related suspected thrombosis. The fresh clot may be not visualized and the diagnosis done on the presence of a vein enlarged and rigid, without changes on respiratory phases or respiratory maneuvers. Useful findings to rule-out thrombosis are an echo-free compressible vein, normal response to respiratory maneuvers-vein collapse on brief deep inspiration (sniff test), and enlargement on Valsalva test normal color Doppler and biphasic spectral display on duplex sonography [34–37]. The main obstacle for the diagnosis of UEDVT is the presence of overlying bones on the medial subclavian vein and centrally located veins, innominate and superior vena cava, that makes them difficult to visualize and impossible to directly assess by compression techniques.

Spectral Doppler abnormalities in the subclavian vein may be predictable for central occlusions. Flow void on color Doppler and a dampened nonpulsatile and nonphasic flow on duplex examination are diagnostic for a central venous thrombosis [38]. A reversed flow in the jugular vein may indicate thrombosis in the innominate vein with the internal jugular vein serving as a collateral pathway. Patel et al. [34] related a 100% positive predictive value and 91% negative predictive value for sonography in the diagnosis of complete central occlusions. Small nonobstructive thrombus may remain undiagnosed and large collateral veins misinterpreted as a normal vein, leading to false negative results. To overcome some of the limitations of US examination of the upper limb veins, a small footprint sector transducer from a supraclavicular or suprasternal approach may be of aid. CDDUS is a reliable method for diagnosing CVC-related thrombosis of the upper limb veins especially if several parameters are evaluated in combination [39]. High diagnostic accuracy of UEDVT was found in 6 prospective studies, with a sensitivity of 78–100% and a specificity of 82–100% [8, 40–44]. False positive results were unusual. A sensitivity of 100% and a specificity of 94% for compression US and color Doppler US for UEDVT using venography as the reference test ware reported by Prandoni et al. [44].

Chronic thrombosis in a patient with long-term catheterization is more challenging, as enlargement of the thrombotic lumen is not present. Color Doppler is even more useful in chronic thrombosis detecting collateral veins and an echogenic, flow void, and small caliber central vein. Large veins in an unusual anatomic position and without the accompanying artery must be recognized as enlarged collaterals and not be mistaken for the main vein. Aliasing due to high velocities and high pulsatility in the stenosed areas in comparison to dampened peripheral waveforms are additional diagnostic parameters. Frozen valve leaflets and echogenic synerchias may be seen as sequels of previous thrombosis [35, 37, 45]. In any case, the diagnosis of catheter-associated deep venous thrombosis may be difficult. Doppler ultrasound has a lower accuracy in this setting than it does in lower extremity venous thrombosis [46].

A particular different issue is acute on chronic thrombosis. The enlarged vein with hypoechoic lumen represents an acute process. Recurrent thrombosis is a challenging diagnosis for all imaging modalities. Comparison with a baseline examination may be helpful in these cases.

**Figure 4:**
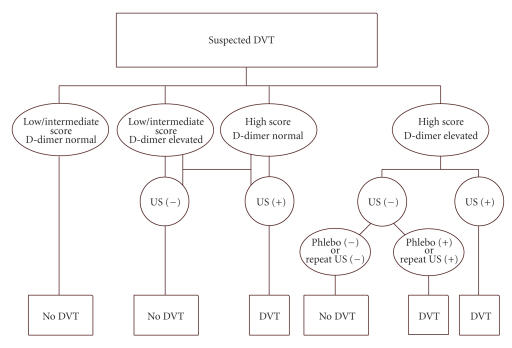
Algorithm for the diagnosis of DVT in symptomatic patients. By applying a diagnostic strategy based on the clinical score and D-dimer test; venous USD is performed in patients with a high clinical score, an elevated D-dimer, or both. The appropriate examination is compression color duplex ultrasound of the complete venous system, including the distal veins, when focal symptoms or physical findings are present and bilateral examination in the high-risk patient. Contrast venography is reserved for a minority of cases. Modified from Mantoni M. Ultrasound of limb veins. Eur Radio 11 : 1557-62, 2001 (with author's permission).

The clinical diagnosis of DVT is unreliable, but clinical prediction rules based on signs and symptoms do facilitate the categorization of patients into high, low, or medium risk categories [47]. A diagnostic strategy combining clinical score, D-dimer test, and compression US may refine the selection of patients. D-dimer assays have a high negative predictive value in patients with suspected VTE and can exclude the diagnosis. Based on clinical score and D-dimer test, venous US will be performed in patients with a high clinical score, an elevated D-dimer, or both (Figure 4).

Screening patients with plasma D-dimer and ultrasonography of the lower limbs may be the most cost-effective strategy. Ascending venography is reserved for patients with negative or equivocal CDDUS results and a high clinical probability of DVT [28, 48–50]. In the current state of the art, CDDUS is the modality of choice for the diagnosis of DVT. The appropriate examination is compression color duplex ultrasound of the complete venous system, including the distal veins when focal symptoms or physical findings are present and a bilateral examination in the high-risk patient. It is an accurate examination and allows an early and safe diagnosis of thrombosis without straining the patients. It is the main diagnostic tool in symptomatic patients and in screening asymptomatic DVT in specific high-risk populations. Pitfalls and limitations of venous ultrasound are related to veins anatomy, flow changes, technical limitations, and operator expertise.

## 4. COMPUTERIZED TOMOGRAPHY ANGIOGRAPHY AND VENOGRAPHY

Multidetector CTA, combined with venous-phase imaging (CTA-CTV), can accurately diagnose a pelvic vein or inferior vena cava occlusion, sometimes the source of significant pulmonary emboli. Multidectector helical CT (MDCT) of the chest (100–140 mL of contrast medium injected at a rate of 3 mL/s) is followed by venous-phase imaging CT of the lower limbs without any additional contrast medium injection [51]. Indirect MDCT venography is acquired from the upper calves to the mid-abdomen. Thrombosis appears as a hypodense mass sometimes encircled by the hyperdense rim of contrast medium. The reported specificity and sensitivity compared with ultrasound is variable [52]. Coche et al. [51] compared the results of CT venography for diagnosing DVT with those of Doppler sonography and phlebography or repeated focalized sonography in case of discrepancy. Sensitivity and specificity of CTV were 93% and 97%, respectively (kappa = 0.88). CT venography in addition to CT pulmonary angiography is a relatively accurate method for evaluation of femoropopliteal venous thrombosis. In a comparative study between CTA-CTV and sonography, Garg et al. [53] found a 100% sensitivity, 97% specificity, 100% negative predictive value, and 71% positive predictive value for CTV. Satisfactory or good quality CT venography examination was obtained in 97% of the studies. Two CT venography studies had false-positive findings due to flow artifacts. The authors concluded that combined CT pulmonary angiography and CT venography may be more efficacious than sonography or two separate examinations in the selected patients. In another trial, CT venography had 93% accuracy compared with sonography in identifying deep venous thrombosis. However, the positive predictive value of CTV was only 67%, suggesting that sonography should be used to confirm the presence of isolated DVT before anticoagulation is initiated. CT venography interpretation should be performed with knowledge of certain pitfalls [54].

The prospective investigation of pulmonary embolism diagnosis II trial was conducted to investigate the accuracy of MDCTA alone and combined with venous-phase imaging (CTA-CTV) for the diagnosis of acute pulmonary embolism [55].

MDCTA alone had 83% sensitivity, 96% specificity, and positive predictive value with a concordantly high or low probability on clinical assessment. CTA-CTV for PE had 90% sensitivity and 95% specificity and was nondiagnostic with a discordant clinical probability like MDCTA alone. Missing diagnoses were due to poor image quality of either CTA or CTV. According to this trial, MDCTA-CTV has a higher diagnostic sensitivity than does CTA alone with similar specificity in patients with suspected PE. The predictive value of both of them is high with a concordant clinical assessment, but additional testing is necessary when the clinical probability is inconsistent with the imaging results. According to Cham et al. [56], a substantial number of patients suspected to have PE had DVT in the absence of PE. The combined technique of pulmonary CTA-indirect CTV has been shown to identify DVT in up to 18% of patients with suspected PE who have no evidence of emboli on CTPA and thus could have a significant effect on patient care. Indirect MDCT venography is as accurate as sonography in the diagnosis of femoropopliteal DVT and can further reveal thrombus in large pelvis veins and the inferior vena cava, an important advantage over sonographic screening for DVT [57], although the technique is slightly more time consuming (up to 4 min delay after contrast injection) and has an increased radiation dose [58].

## 5. MAGNETIC RESONANCE VENOGRAPHY

Two-dimensional time-of-flight venography (TOF-MRV) is the technique of choice for magnetic resonance venography. Studies may be performed without contrast and can depict emboli as filling defects or directly detect the thrombus. MR direct thrombus imaging (MR-DTI) is a novel technique which detects metahemoglobin, allowing direct visualization of pulmonary emboli and simultaneous imaging of the legs without the need for intravenous contrast. This technique uses a T1-weighted gradient-echo sequence, with a preexcitation radio-frequency pulse to abolish fat signal, and an inversion recovery time chosen to nullify signal from flowing blood to maximize thrombus conspicuity. The technique is 98% sensitive and 96% specific for diagnosing DVT when compared with ultrasound and contrast venography. Early data suggest that MR-DTI is also highly accurate in detection of PE and the safety of withholding treatment on the basis of MR-DTI alone is currently being evaluated [59]. Acute occlusion of the pelvic veins and the inferior vena cava, often due to extension from the femoropopliteal system, represents a major risk for PE. Color flow Doppler imaging is often limited for the diagnosis of iliocaval thrombosis owing to obesity and bowel gas. Both CT scans and MR imaging can accurately diagnose acute pelvic vein or inferior vena cava occlusion and are as well helpful in diagnosing central chest vein occlusion. MRI is preferred because it is noninvasive, does not require contrast agent, carries no exposure to ionizing radiation, that is definitively demanded for pregnant women, and is highly accurate and reproducible [60].

Furthermore, MRV can differentiate an acute occlusion from chronic thrombus. In a study designed to evaluate the diagnostic value of MRV and color Doppler US in the assessment of DVT compared with contrast-enhanced venography, MRV was 100% sensitive and 100% specific in the diagnosis of DVT above the knee. Color Doppler imaging depicted 13 of 15 cases of DVT and 5 of 6 venous examinations that had normal results, yielding sensitivity and specificity of 87% and 83%, respectively. The differences in sensitivity and specificity between MRV and color Doppler US were not statistically significant [61]. In a recent meta-analysis to estimate the diagnostic accuracy of MRV for DVT, the pooled estimate of sensitivity was 91.5% (95% CI: 87.5–94.5%) and the pooled estimate of specificity was 94.8% (95% CI: 92.6–96.5%). Sensitivity for proximal DVT was higher than sensitivity for distal DVT (93.9% versus 62.1%) [62]. MR venography seems to be more accurate than color Doppler sonography in detecting the extension of deep venous thrombosis. Shankar et al. [63] performed two-dimensional gated inflow and phase contrast MRV in children with suspected upper extremity CVC-related thrombosis, to assess the extent of venous thrombosis and to locate patent veins for replacement central venous catheter. MRV was more accurate than Doppler ultrasonography and contrast studies for defining the extent of venous thrombosis. MRV correctly showed venous anatomy and patency for reinsertion of CVC. MRV is considered medically indicated for evaluation of venous thrombosis or occlusion in the large systemic veins (e.g., superior vena cava, subclavian, or other deep veins in the chest), for differentiation of tumor thrombus and blood clot and diagnosis of superior vena cava syndrome. The peer reviewed medical literature has not established MRV to be superior to duplex ultrasonography for diagnosis of deep vein thrombosis in the arms or legs. MRV has not been shown to be superior to US for lower limb DVT, except in imaging the deep femoral and hypogastric vessels. However, information about these vessels is not needed for management decisions, except in patients with pulmonary emboli where the source of the emboli has not been identified by ultrasonography [64]. MRV has the potential to be used as a stand-alone test for DVT but requires further evaluation. Therefore it is considered to be experimental and investigational for this application. Due to its high cost and limited availability, MRV should be reserved to diagnose DVT in patients for whom ultrasound examination is inappropriate or unfeasible [62] and to replace venography and CTV in pregnant women and patients with contraindications to iodinated contrast media injection.

## 6. NUCLEAR MEDICINE VENOGRAPHY

The radionuclide investigation of DVT includes such techniques as radionuclide venography and thrombus-avid scintigraphy. Although these methods have not been as thoroughly evaluated as compression ultrasound, studies thus far have indicated encouraging results, and further investigations are warranted [65]. Radionuclide venography of the upper extremity has been described as a reliable noninvasive procedure for early diagnosis of upper limb venous thrombosis associated with indwelling CVC. It is performed by injecting both arms with approximately 5 mCi of technetium pertechnetate followed by a normal saline flush. The dynamic images are acquired on a large field of view camera with a high-energy low-resolution collimator at the rate of two frames per second [66, 67]. (99 m)TC-MAA radionuclide imaging is a useful method for noninvasive detection of DVT and PTE. Combined radionuclide venography and perfusion lung scan can also be performed in the same setting if Tc99m-MAA is used [68]. The radionuclide venogram appears accurate in the proximal veins and in excluding but not diagnosing distal venous thrombosis. The potential advantages of radionuclide venography versus contrast venography are low-volume and low-flow injection, no need to access a large peripheral vein, no adverse side effects, low radiation exposure (130 mrads), rapidity of execution, and no patient preparation. The disadvantage is the low anatomic detail [66, 67].

In summary, invasive testing for venous thromboembolism can be safely avoided in the majority of patients, using diagnostic strategies combining noninvasive tests.

Color and duplex ultrasound with manual compression (CDDUS) is the most sensitive and specific noninvasive test and is nowadays accepted as the modality of choice for the diagnosis of DVT. CT venous-phase imaging at the time of CT pulmonary angiography and MR venography is comparable with venous ultrasonography in the evaluation of femoropopliteal DVT. The iliac veins and vena cava, vessels poorly shown on ultrasonography but sometimes the source of significant pulmonary emboli, are also depicted by CT and MR venography. MRV can differentiate an acute occlusion from chronic thrombus. Due to its high cost and limited availability, MRV is not used for the routine diagnosis of DVT and should be reserved for the examination of inaccessible veins on ultrasonography and as a complementary test in nondiagnostic ultrasound studies for pregnant women and patients with contraindications to iodinated contrast media injection. Studies on venous scintigraphy have indicated encouraging results but further investigations are warranted. A diagnostic strategy combining clinical score, D-dimer test, and compression US can be used in a systematic way to reliably rule in or exclude venous thromboembolism.

## 7. CONCLUSIONS

Due to its high specificity, a negative examination may preclude anticoagulant treatment. A strategy combining clinical score and D-dimer test refines the selection of patients. Phlebography is the gold standard method but is invasive and carries risks of contrast media complications and ionizing radiation. CTV following pulmonary CTA and MRV is useful to detect iliocaval thrombosis. MRV can differentiate acute from chronic thrombosis and diagnose central obstructions. RNV has low-anatomic detail.

CDDUS is the modality of choice for the diagnosis of DVT. A diagnostic strategy combining clinical score, D-dimer test, and CDDUS is recommended. Phlebography is reserved for discrepant noninvasive studies.
